# Distinct activity of the bone-targeted gallium compound KP46 against osteosarcoma cells - synergism with autophagy inhibition

**DOI:** 10.1186/s13046-017-0527-z

**Published:** 2017-04-12

**Authors:** Bernd Kubista, Thomas Schoefl, Lisa Mayr, Sushilla van Schoonhoven, Petra Heffeter, Reinhard Windhager, Bernhard K. Keppler, Walter Berger

**Affiliations:** 1 Department of Orthopedics, Medical University of Vienna, Waehringerguertel 18-20, A-1090 Vienna, Austria; 2 Department of Medicine I, Institute of Cancer Research and Comprehensive Cancer Center, Medical University Vienna, Borschkegasse 8a, A-1090 Vienna, Austria; 3 Research Platform “Translational Cancer Therapy Research”, University Vienna and Medical University Vienna, Vienna, Austria; 4 Institute of Inorganic Chemistry, University of Vienna, Waehringerstr. 42, A-1090 Vienna, Austria

**Keywords:** Osteosarcoma, KP46, Anticancer gallium compound, Autophagy, Obatoclax

## Abstract

**Background:**

Osteosarcoma is the most frequent primary malignant bone tumor. Although survival has distinctly increased due to neoadjuvant chemotherapy in the past, patients with metastatic disease and poor response to chemotherapy still have an adverse prognosis. Hence, development of new therapeutic strategies is still of utmost importance.

**Methods:**

Anticancer activity of KP46 against osteosarcoma cell models was evaluated as single agent and in combination approaches with chemotherapeutics and Bcl-2 inhibitors using MTT assay. Underlying mechanisms were tested by cell cycle, apoptosis and autophagy assays.

**Results:**

KP46 exerted exceptional anticancer activity at the nanomolar to low micromolar range, depending on the assay format, against all osteosarcoma cell models with minor but significant differences in IC_50_ values. KP46 treatment of osteosarcoma cells caused rapid loss of cell adhesion, weak cell cycle accumulation in S-phase and later signs of apoptotic cell death. Furthermore, already at sub-cytotoxic concentrations KP46 reduced the migratory potential of osteosarcoma cells and exerted synergistic effects with cisplatin, a standard osteosarcoma chemotherapeutic. Moreover, the gallium compound induced signs of autophagy in osteosarcoma cells. Accordingly, blockade of autophagy by chloroquine but also by the Bcl-2 inhibitor obatoclax increased the cytotoxic activity of KP46 treatment significantly, suggesting autophagy induction as a protective mechanism against KP46.

**Conclusion:**

Together, our results identify KP46 as a new promising agent to supplement standard chemotherapy and possible future targeted therapy in osteosarcoma.

**Electronic supplementary material:**

The online version of this article (doi:10.1186/s13046-017-0527-z) contains supplementary material, which is available to authorized users.

## Background

Osteosarcoma (OS) is the most common primary malignant bone tumor. Because of a high rate of metastatic spread, curative treatment with surgery alone is rare. The long-term survival of patients with OS has improved during the last 40 years from 20% to nearly 80% due to the use of neoadjuvant chemotherapy in combination with surgery [1]. However 20% of patients without evidence of metastasis at diagnosis either relapse locally or develop systemic disease preferentially by metastasis to the lungs or, less frequently, distant bones [2]. This disease progression is primarily related to poor chemotherapy response resulting in a very unfavourable prognosis [1, 3]. So far, histological response to neo-adjuvant chemotherapy is the most reliable predictor of survival in non-metastatic OS patients. Common chemotherapeutic regimens in OS include methotrexate, doxorubicin and cisplatin. However, if the tumor does not respond well to initial standard chemotherapy, no real alternative treatment option exists so far. Moreover, several previous studies demonstrated that a more aggressive and intensified chemotherapy could only improve histological tumor response but failed to improve patient survival [4]. Therefore the implementation of new, preferentially bone-targeting drugs that can overcome chemotherapy resistance and inhibit metastasis especially to the lung are highly desirable and could further increase survival in OS patients [5].

The antitumor activity of several gallium, germanium, titanium and ruthenium compounds has been recognized for some time, but none of these compounds have reached clinical routine so far [6, 7]. In particular, simple gallium salts (gallium nitrate and gallium chloride) have been evaluated for their safety and efficacy in several clinical studies [8]. Despite pronounced single-agent activity in lymphomas and bladder cancer and evidence for synergistic effects in combinations with established drugs, unfavorable pharmacokinetic and toxicological properties have prevented their routine use. Consequently, an organometallic gallium complex - KP46 (tris(8-quinolinolato)gallium(III)- with oral bioavailability was developed and has reached clinical evaluation [9]. KP46 is 10-times more active than gallium chloride in vitro and has been reported to circumvent different forms of drug resistance [10]. The great affinity of gallium and KP46 for bone [11] clearly suggests that this gallium compounds might bear the potential to treat malignant bone tumors like OS. The effects of gallium salts on human cells are numerous and various [8]. It has been widely accepted that a main antitumor activity is based on the inhibition of the iron-dependent enzyme ribonucleotide reductase. Due to its ability to compete iron uptake, gallium salts and also KP46 affects intracellular iron pools, but may also interact directly with ribonucleotide reductase displacing iron from the R2 subunit of this enzyme [12, 13]. Additionally, gallium modifies the three-dimensional structure of DNA and blocks replication, modulates protein synthesis, and inhibits the activity of other enzymes, such as ATPases, DNA polymerases, and tyrosine-specific protein phosphatase. Furthermore, antimitotic effects of gallium have been described [14]. Gallium complexes with lipophilic ligands - such as KP46 - have been developed to improve intestinal absorption without altering the pharmacodynamic effects [11]. KP46 has already been successfully tested in a phase-I clinical trial on renal cell cancer and the evaluation of KP46 in a phase-II clinical trial was recommended [15].

Proteins of the Bcl-2 family play an important role in regulation of programmed cell death and are possible future candidates for targeted therapy in OS. Recent studies demonstrated that gallium-induced cell death in lymphoma but also cytotoxic activity of KP46 against lung and colon cancer cells might involve pro-apoptotic Bcl-2 members like Bax and Bim triggering the mitochondrial apoptotic pathway [16, 17]. Interestingly, increased expression of the anti-apoptotic Bcl-xL was associated with poorer survival of OS patients [18] while Bcl-xL inhibition significantly enhanced chemo- and radiosensitivity of OS cells in vitro [19]. Together these data suggest that inhibition of anti-apoptotic Bcl-2 members might be a feasible strategy to sensitize OS cells against chemotherapy.

Considering this information, aim of this study was to evaluate the possible antitumor effect of the bone-targeting gallium complex KP46 against human OS cells in vitro and to gain insight into the underlying molecular mode-of-action. Additionally, we set out to evaluate possible synergistic effects of KP46 with standard chemotherapeutics and compounds targeting apoptosis inhibition by members of the Bcl-2 family.

## Methods

### Chemicals

The organometallic gallium compound KP46 (tris(8-quinolinolato)gallium(III) was synthesized at the Institute of Inorganic Chemistry, University of Vienna (Vienna, AT). Doxorubicin, methotrexate (MTX), chloroquine, bafilomycin A1 and cisplatin were purchased from Sigma (St. Louis, MO), and obatoclax mesylate (GX15-070) from Selleck Chemicals Inc. (Houston, TX). Cisplatin was dissolved to a 5 mM stock in dimethylformamide (DMF, Sigma, St. Louis, MO), MTX to a 10 mM stock in NaOH and all other substances to 4–10 mM stocks in dimethylsulfoxide (DMSO, Sigma, St. Louis, MO). Stock solutions were diluted into culture medium immediately before use to obtain indicated concentrations. Final concentrations of solvents (DMSO, DMF, NaOH) were always less than 1% and tested for anti-OS activity in parallel.

### Cell culture

OS cell lines MG-63, HOS, Saos-2 and U-2 OS were obtained from the American Type Culture Collection (ATCC, Manassas, VA). The human lung fibroblast (HLF) culture was established from a non-malignant lung surgery specimen. Cells were cultured in the respective growth media (Saos-2 in Mc Coy’s 5A medium, U-2 OS in Iscove’s Modified Dulbecco’s Medium and all other cell lines in RPMI1640 medium) supplemented with 10% fetal calf serum (FCS), obtained from PAA, Pasching, Austria, at 37 °C in 5% CO_2_ and regularly checked for *Mycoplasma* contamination.

### Cell viability assay

Cells were seeded (2 × 10^4^ cells/ml) in 100 μl growth media per well in 96-well plates. After a recovery period of 24 h, cells were treated with the indicated concentrations of the investigated drugs added to the cells in another 100 μl growth medium. If not indicated otherwise, drug exposure time was always 72 h. Cell viability was measured by the 3-(4, 5-dimethylthiazol-2-yl)-2,5-diphenyltetrazolium bromide (MTT)-based vitality assay (EZ4U; Biomedica, Vienna, Austria) following the manufacturer’s recommendations. Cytotoxic effects were calculated with Graph Pad Prism software 5.0 (using a point-to-point function) (La Jolla, USA) and were expressed as IC_50_ values calculated from full dose-response curves (drug concentrations inducing a 50% reduction of cell number in comparison to untreated control cells cultured in parallel). Values given are derived from at least three experiments performed in triplicates. Drug interactions in combination experiments were estimated using CalcuSyn software (Biosoft, Ferguson, MO) as described [20, 21] and expressed by the combination index (CI) with CI < 0.9 representing synergism, CI 0.9–1.1 additive effects and CI > 1.1 antagonism.

### Colony formation assay

Cells were plated (1 × 10^3^ cells/ml) in 500 μl in 24-well plates and allowed to recover for 24 h. Drugs were added in 100 μl growth medium as indicated and cells were exposed to drugs for 7 days. After the drug exposure period, cells were washed with phosphate-buffered saline (PBS), fixed with methanol at 4 °C and stained with crystal violet. Clone area/μm^2^ was determined using high-resolution pictures (Nikon7100) of at least 4 wells derived from two independent experiments in duplicate using Image J software. Moreover, single colonies >15 cells ﻿were counted using ImageJ Java software as described [22]. Experiments were performed in duplicate and repeated twice.

### Hoechst 33258/propidium iodide (HOE/PI) staining

OS cell lines were seeded (5 × 10^4^ cells/well) in 24-well plates and exposed to KP46, obatoclax or a combination of both drugs at the indicated concentrations for 24 or 48 h exposure time. After the indicated incubation times, cells were stained with 2 μl/ml Hoechst 33258/propidium iodide mix (HOE/PI; HOE 1 mg/ml in PBS/PI 2.5 mg/ml in PBS), and incubated for at least an hour before microscopical evaluation using a Nikon Eclipse Ti inverted microscope (Vizitron Systems, Germany) [22]. Positive staining with PI indicated dead cells (necrotic or late apoptotic). Nuclei of viable cells exhibited even blue fluorescence based on DNA staining by HOE. Bright blue fluorescence in condensed chromatin of PI-negative cells indicated mitosis characterized by regular mitotic features or early apoptosis based on small condensed nuclei or formation of apoptotic bodies. The number of viable undamaged, mitotic and apoptotic cells were counted in four optical fields per experimental group and well from two experiments in duplicate (ImageJ, Java Software) to quantify the photomicrographs.

### Cell migration assays

Twenty-four-well plates were filled with 800 μl growth medium with 10% FCS, inserts (cell culture insert for 24-well plates, 8.0 μM pore size, Falcon™ ThermoFisher Scientific) were placed in wells and filled with 300 μl cell suspension (1 × 10^5^ cells/ml) in growth medium without FCS. Drugs were added to the insert and well underneath. After 24 h drug exposure and migration time, inserts were removed. Lower wells were incubated with fresh culture medium for an additional 7 days, washed with PBS, fixed with methanol and stained with crystal violet. Wells and inserts were photographed and cell clones counted as mentioned above for the clonogenic assay.

### Analysis of autophagy by Western blot

OS cells were seeded, proteins extracted after 24 h drug exposure and processed for Western blotting as described [22]. Microtubule-associated protein 1 light chain 3 (LC3B), ATG5, ATG7 and Beclin-1 primary antibodies were purchased from Cell Signaling Technology (Danvers, USA). Monoclonal mouse antibody β-actin was obtained from Sigma. All primary antibodies were used as 1:1000 working dilutions and incubated overnight at 4 °C. Horseradish peroxidase-conjugated secondary antibodies [23] (Santa Cruz Biotech, Dallas, USA) were used as 1:10.000 working dilutions. Autophagic flux was determined based on the method published by Chittaranjan et al. [24]. In short, the lowest bafilomycin A1 dose inducing maximal LC3-II accumulation was determined by dose-response analyses and found to be 5 nM in both cell lines analysed (HOS, U-2 OS). Then KP46 at 1 and 10 μM was combined with bafilomycin (5 nM), obatoclax (250 nM) or both for 24 h and alterations in autophagic protein expression and LC3-I cleavage determined. Densitometric analysis of Western blot bands was performed using Image J software as published [23] and protein expression quantified relative to β-actin in all cases. At least three Western blots from two independent experiments were analysed.

### Acridine orange (AO) staining

Autophagosomes were stained with acridine orange (AO; 1 μg/ml), Merck, Darmstadt, Germany) after 24 h drug exposure on confluent cells (2 × 10^5^ cells/ml). Stained cells were observed under the microscope and microphotographs taken (Nikon Eclipse Ti, Life-Cell Imaging) before dilution in FACS-PBS. Flow cytometry was performed using LSRFortessa flow cytometer and data analyzed with Flowing Software (University of Turku, Finland) [25]. Fluorescence intensity was measured with the following filter settings: FL3 – red (488 nm/670 nm) and FL1 –green (488 nm/533 nm). The volume of the acidic compartment was estimated by the fluorescence intensity ratio FL3/FL1.

### Cell cycle analysis

Cells (2 × 10^5^ cells/well) were incubated for 24 h with indicated concentrations of KP46 or obatoclax in 6-well plates at 37 °C. After drug exposure, cells were collected and fixed in 70% ethanol at −20 °C for 1 h. RNase (0.2 mg/ml, Sigma, St. Louis, MO) and PI (0.01 mg/ml, Sigma, St. Louis, MO) diluted in FACS-PBS were used to degrade RNA and stain DNA, respectively. Cell cycle progression was examined by flow cytometry using FACS Calibur (Becton Dickinson, Palo Alto, CA) as described in Hoda et al. [26]. CellQuest Pro software (Becton Dickinson) was used to analyze the resulting DNA histograms.

### Statistical analysis

Data are presented as means ± SD of at least three experiments performed in triplicate, unless stated otherwise. Statistical significance between treatment groups was calculated with Graph Pad Prism 5.0 using *t*-test or one-way analysis of variance (ANOVA) including Bonferroni post-tests as appropriate. P-values below 0.05 were considered as statistically significant (* *p* < 0.05; ** *p* < 0.01; *** *p* < 0.001).

## Results

### KP46 exerts potent antitumor activity in OS cell lines

**Fig. 1:**
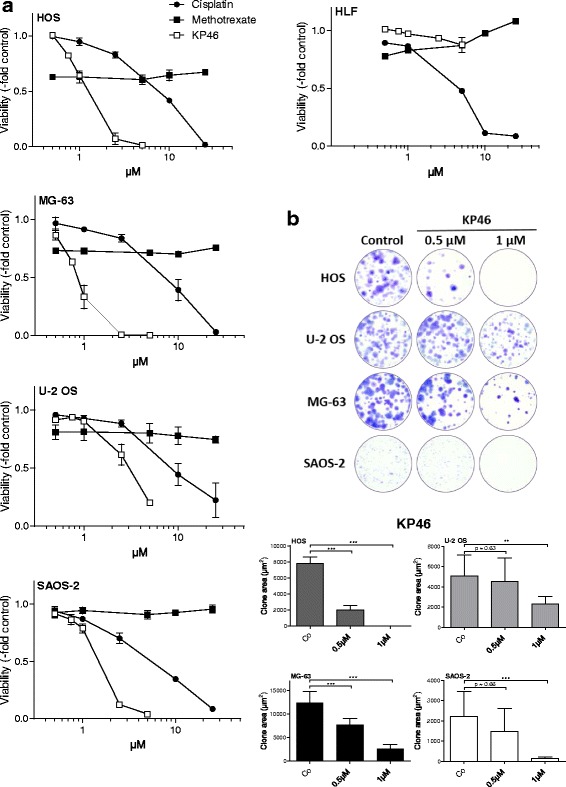
The anticancer gallium compound KP46 is highly active against OS cells. **a** Full dose response curves for KP46 as compared to the standard OS chemotherapeutics cisplatin and methotrexate were established from 72 h continuous drug exposure assays in the indicated OS cell lines and non-malignant HLF cells as indicated. One out of three independent experiments performed in triplicate is shown. For mean IC_50_ values derived from all three experiments, compare Table 1. **b** The impact of low-dose KP46 on the clonogenic potential of the indicated OS cell lines was determined as clonal area (compare Material and Methods). High resolution micrographs of crystal violet stained 24-well plates (*upper panel*) were taken and evaluated Image J software (*lower panel*). Data are derived from two independent experiments performed in duplicates. Student’s *t*-test; ** *p* < 0.01; *** *p* < 0.001

The anti-OS activity of KP46 was tested in two assay formats with different exposure times, namely standard MTT tests after 72 h and clonogenic assay after 7 days drug exposure (Fig. 1a and b, respectively). KP46 was active against all four tested OS cell lines with minor but significant differences in potency. IC_50_ values derived from the MTT format ranged from 1.20 μM in the most sensitive MG63 to 3.83 μM in the most resistant U-2 OS cell line (Fig. 1a, Table 1). Hence, KP46 was active at lower concentrations as compared to the routinely used OS drugs cisplatin and methotrexate (Fig. 1a and Table 1). To demonstrate a possible tumor-specific activity of KP46, the impact on non-malignant fibroblasts (HLF) cell cultures was compared. Interestingly, not any sign of reduced viability was seen up to 5 μM and the IC_50_ value was 9.01 μM suggesting enhanced activity of KP46 towards tumor cells. In contrast cisplatin exhibited the strongest activity in case of non-malignant HLF cells (Fig. 1a, Table 1).

**Table 1:** Activity of KP46 and obatoclax as compared to standard OS chemotherapeutic agents

Cell Line	KP46 (μM)	Cisplatin (μM))	Methotrexate (μM)	Doxorubicin (nM)	Obatoclax (nM)
	IC50 values (mean ± SD)				
MG-63	1.204 ± 0.392	7.055 ± 1.6	>50	111.8 ± 87.6	102.9 ± 19.9
HOS	1.210 ± 0.344	6.732 ± 2.5	>50	77.6 ± 16.2	65.1 ± 12.8
U-2 OS	3.830 ± 1.232	8.169 ± 3.2	>50	162.5 ± 123.7	306.7 ± 171.9
SAOS-2	1.641 ± 0.113	5.277 ± 2.1	>50	100.6 ± 4.1	74.0 ± 6.3
HLF	9.01 ± 0.012	3.919 ± 1.2	>50	>250	154.6 ± 15.0

### KP46 induces OS cell death and minor changes in cell cycle distribution

In the long-term expose assay, KP46 significantly reduced the clonogenic potential in all four OS cell lines already at a dose of 1 μM (mean clone area shown in Fig. 1b). In these experiments, the differences in KP46 sensitivity became more obvious, with distinct reduction of clone formation already in the nanomolar range in the more sensitive cell lines HOS and MG-63, while clone size was not significantly reduced at this concentration in the more resistant U-2 OS and SAOS-2 cells.

**Fig. 2:**
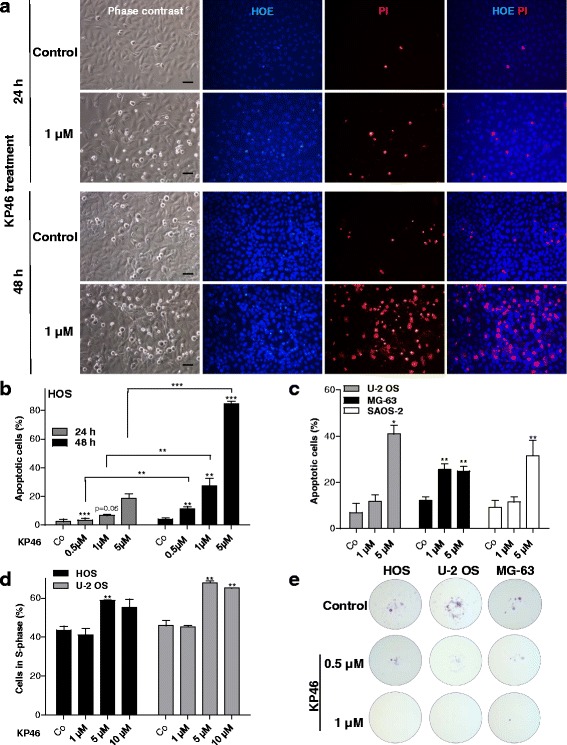
Impact of KP46 on OS cell viability, cell cycle distribution and migratory potential. **a**, **b** Induction of apoptotic/necrotic cell death was determined by concomitant staining with HOE/PI as described in Methods. Photomicrographs of HOS cells treated for 24 and 48 h are representatively shown in (**a**; size bar, 50 μm) and opposed to the microscopical evaluation of two experiments in duplicate for HOS (**b**) and the other OS cell lines (**c**) at the indicated times and concentrations of KP46 treatment. Stars directly above the bars define significance compared to the solvent controls, whereas significance between 24 and 48 h of treatment are indicated by brackets. **d** The effect of a 24 h KP46 exposure on cell cycle distribution in the indicated OS cells was measured by FACS analysis and the percentage of cells in S-phase is shown as mean of three independent experiments. **e**) Impact of low-dose KP46 on cell migration was measured in transwell filter assay. After a 24 h migration period under KP46 exposure, surviving cells in the lower well were allowed to form colonies in drug-free medium for 7 days. Colonies were fixed and stained at day 10 after start of the migration period. One-way analysis of variance (ANOVA) (**b**, **c**) and student’s *t*-test (**d**); * *p* < 0.05; ** *p* < 0.01; *** *p* < 0.001

To estimate cytotoxic versus cytostatic effects, OS cell cultures treated with KP46 were stained with HOE/PI to indicate early and late apoptosis and necrosis (HOS at 24 and 48 h drug exposure in Fig. 2a as microphotographs and respective evaluation in Fig. 2b; all other cell lines in Fig. 2c). KP46 treatment of HOS cells resulted in a rapid and dose-dependent increase of rounded, detached cells (phase contrast images for HOS and SAOS-2 cells in Additional file 1: Figure S1). Interestingly only a subpopulation of the detached cells (especially at 24 h exposure) harbored condensed DNA as characteristic features of apoptotic cell death (Fig. 2a, b). Conversely, a proportion of PI-positive and hence dead cells lacked any signs of chromatin condensation suggesting that also a form of rapid non-apoptotic cell death occurs in addition to apoptosis. Nevertheless, the amount of apoptotic cells was increased by KP46 in all cell lines at 5 μM already within 24 h (Fig. 2c).

Concerning impact on cell cycle progression, only a minor increase of the S-phase fraction from about 5 μM KP46 onwards was observed (Fig. 2d). Additionally, changes in cell cycle distribution did not correlate with the IC_50_ values. Together these data suggest that the anti-OS activity of KP46 is more based on cytotoxic than cytostatic effects.

### KP46 inhibits OS cell migration

Migration-based metastasis strongly contributes to aggressiveness of OS [3, 27]. To investigate the impact of KP46 on cell motility, we conducted trans-well migration assays with the four investigated OS cell models (Fig. 2e). SAOS-2 cells were unable to migrate to the lower chamber and form colonies at the lower well. The three other cells readily formed colonies at the lower chamber within 7 days when regular growth media were used. Addition of subtoxic 0.5 μM KP46 already significantly reduced and 1 μM KP46 completely blocked trans-well cell migration of all investigated OS models.

### Combination of KP46 with standard chemotherapeutics

**Fig. 3:**
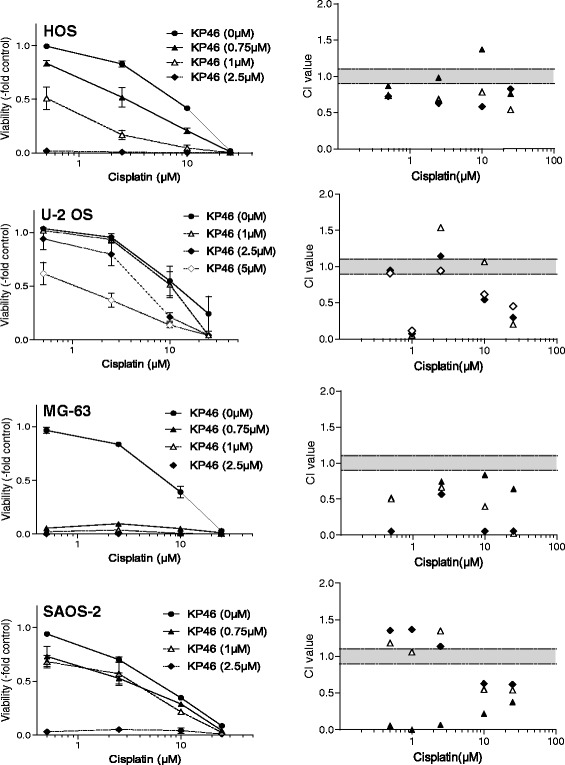
The gallium compound KP46 widely synergizes with the standard OS chemotherapeutic agent cisplatin. The two compounds were applied either alone or in combination at the indicated concentrations for 72 h continuous drug exposure. Cell viability was determined by an MTT-based survival assay. Dose-response curves at the left are compared to the respective combination indices (CI) calculated by CalcuSyn Software at the right panels. CI < 0.9 represents synergism, >1.1 antagonism and 0.9–1.1 (*grey area*) additive effects

To investigate the interaction of KP46 with clinically used OS chemotherapeutics, drug combination experiments were performed. While with methotrexate and doxorubicin mainly additive or even antagonistic effects were found (data not shown), the combination with cisplatin was widely synergistic in all investigated cell models (Fig. 3). Interestingly, the synergistic effects - obvious from the CI values <0.9 in the right panels of Fig. 3 - were not dependent on the single drug KP46 sensitivity of the particular OS cell lines but - at least at certain dosages - observed in all investigated cell models.

### The anti-OS activity of KP46 synergizes with obatoclax

**Fig. 4:**
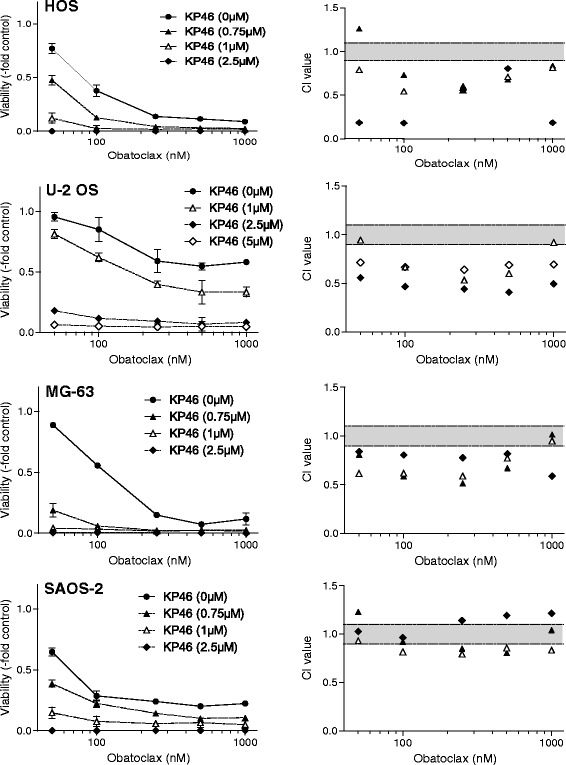
The anti-apoptotic bcl-2 family and autophagy inhibitor obatoclax enhances the activity of KP46 in the majority of the tested OS cell models. The two compounds were either applied alone or in combination at the indicated concentrations for 72 h continuous drug exposure. Cell viability was determined by an MTT-based survival assay. Dose-response curves at the left are compared to the respective combination indices (CI) calculated by CalcuSyn Software at the right panels

In addition to standard chemotherapeutics, we also evaluated combinations of KP46 with several novel “targeted” anticancer agents with a focus on apoptosis-modulating compounds (data not shown). The highest synergistic effects were observed for combinations with the anti-apoptotic Bcl-2 family inhibitor obatoclax (Fig. 4). This synergism was found in all investigated cell models with the exception of SAOS-2. Surprisingly, however, combination of KP46 with the more Bcl-2-specific inhibitor venetoclax (ABT-199) was by far less synergistic as compared to obatoclax (data not shown). Hence, a major contribution of Bcl-2 inhibition in the synergism of KP46 with obatoclax was considered unlikely.

### Autophagy inhibition by obatoclax is underlying the synergism with KP46

**Fig. 5:**
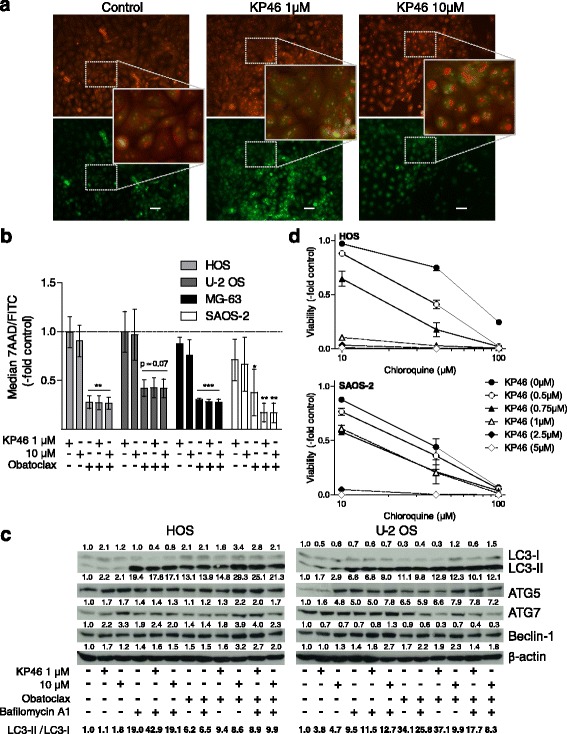
KP46 treatment induces autophagy and synergizes with downstream autophagy inhibitors. **a** HOS cells treated with KP46 for 24 h at the indicated concentrations were stained with AO and fluorescence images taken in the *green* (FL1) and deep *red* (FL3) channel. KP46 induces the formation of large acidic vesicles stained in deep red. For combination experiments with obatoclax compare Additional file 2: Figure S2B. Size bar, 50 μm. **b** Cells treated with KP46 alone and in combination with the downstream autophagy inhibitor obatoxlax at the indicated concentrations were measured for green and red fluorescence by FACS. *Red* (FL3) to *green* (FL1) fluorescence ratios derived from three independent experiments are given. **c** The impact of KP46 alone and in combination with the downstream autophagy inhibitors obatoclax and bafilomycin (250 nM and 5 nM, respectively, 24 h drug exposure) on the expression of the indicated autophagy regulatory proteins and processing of LC3-I to LC3-II was determined by Western blot. Density of the respective bands was evaluated corrected for β-actin expression. Data are given normalized to the untreated controls. The ratio of LC3-II/LC3-I also as a measure of autophagic flux in the combination setting by the method of Chittaranjan et al. [24] is depicted at the bottom line as indicated. **d** Cell viability after combined treatment of OS cells with KP46 and the autophagy inhibitor chloroquine for 72 h at the indicated concentrations was determined by MTT survival assay. Dose response curves for HOS and SAOS-2 cells derived from two independent experiments in triplicate are shown representatively. For the respective CI values compare Additional file 3: Figure S3. Student’s *t*-test; * *p* < 0.05; ** *p* < 0.01; *** *p* < 0.001

Consequently, we set out to identify the molecular mechanisms underlying the synergism of KP46 with obatoclax. Treatment of OS cells with KP46 as single drug caused rapid vacuolization paralleled by cell detachment and deterioration (Additional file 2: Figure S2A). These morphological changes might point towards the induction of autophagy and accumulation of autophagic vesicles in the cell cytoplasm. To clarify whether KP46 as a single drug indeed alters OS cell autophagy, AO staining was performed (compare Material and Methods) and evaluated microscopically (Fig. 5a) as compared to flow cytometry (Fig. 5b). Indeed, progressive formation of larger acidic vesicular organelles staining deep red with AO was observed in KP46-treated cells in addition to the green fluorescence from labelled nucleic acids (HOS representatively in Fig. 5a). However, despite this increased size of acidic vesicles the ratio of red to green AO fluorescence in FACS analysis was widely unaltered in KP46 treated OS cell (Fig. 5b). Especially in SAOS-2 cells the acidic compartment even tended to be reduced by KP46 being the one cell model not exerting a synergism by combination of KP46 and obatoclax (compare Fig. 4).

Obatoclax is well known to inhibit late stage autophagy based on alkalinization of the lysosomal compartment and consequently inhibition of the autophagic flux [28, 29]. Accordingly, the intrinsic AO-stained acidic vesicle compartment was dramatically reduced by obatoclax as single agent and enhanced formation of deep red autophagic vesicles by KP46 completely inhibited (Fig. 5b and microscopical evaluation of HOS cells in Additional file 2: Figure S2B). Alteration of autophagy by KP46 as a single drug was also suggested by moderately enhanced processing of free LC3B (LC3-I) to phosphatidylethanolamine-bound LC3-II especially in the more resistant U-2 OS cells (see ratio LC3-II to LC3-I at the bottom of Fig. 5c). This effect was accompanied by a minor increase in the levels of Beclin-1 and to a stronger extent ATG5, while ATG7 was strongly enhanced only in KP46-hypersensitive HOS cells. Combination with obatoclax further increased processing of LC3B especially in the sensitive cell model which points towards blockade of KP46-enhanced autophagic flux. Hence, we additionally tested a combination of KP46 with bafilomycin A1 to determine the autophagic flux by the method of Chittaranjan et al. [24]. Maximal LC3-II accumulation by bafilomycin (reached at a dose of 5 nM, data not shown) was further enhanced in presence of KP46 confirming increased autophagic flux by this gallium compound (Fig. 5c). Interestingly, the combination of KP46 with obatoclax also moderately enhanced LC3B processing at both concentrations in the sensitive HOS cells while it was reduced at 10 μM KP46 in the more resistant U-2 OS cells. In the latter cell model, also reduction of ATG7 levels by this combination was distinct.

Summarizing the observations from autophagy analyses and viability tests, a tumor cell-protective function of KP46-mediated autophagy seems very likely. To confirm this hypothesis, we treated OS cells with KP46 in combination with chloroquine, another well-known inhibitor of autophagic flux based on a blocked autophagosome fusion with lysosomes. Indeed, we found a distinctly synergistic effect for this autophagy inhibitor in HOS cells while the one in SAOS-2 cell lacking autophagy induction by KP46 again tended to be primarily additive (Fig. 5d and combination indices CI in Additional file 3: Figure S3).

## Discussion

In previous studies, KP46 demonstrated strong anti-cancer effects against cell lines and xenograft models of different human cancer types including colorectal cancer and melanoma [17, 30, 31]. Furthermore, KP46 accumulates selectively in bone tissues [11] suggesting an inherent tropism for primary and secondary bone tumors including OS. We therefore evaluated the activity of KP46 against a panel of human OS cell lines as single agent and its interaction with clinically used OS chemotherapeutics as well as experimental anticancer drugs. KP46 exerted profound cytotoxicity against all tested OS cell lines in the low micromolar to nanomolar range depending on the assay format and exposure time. Besides induction of cell detachment and death at higher concentrations, treatment with low-dose KP46 additionally reduced OS cell migration and clonogenic survival. In contrast, changes in cell cycle distribution during KP46 treatment were comparably minor and consisted of an accumulation in S-phase as has also been reported in previous studies in other cancer types [30, 31]. Despite a significant difference in the sensitivity of the tested OS cell models against the cytotoxic effect of KP46, all tumor cell lines were distinctly hypersensitive as compared to the non-malignant HLF cell line. This may indicate a specific tumor-targeting effect of KP46 and suggests existence of a therapeutic window for this bone-targeting compound at least at the cellular level. Interestingly, cisplatin exhibited the lowest IC_50_ values in the non-malignant HLF cell model well in agreement with the massive adverse effects by this clinically used anticancer metal drug.

The molecular mechanisms underlying the anticancer activity of KP46 are still not well understood. Some studies - including several from our group - suggested apoptotic cell death induction as one major mode of action for KP46 especially after longer drug exposure times [13, 30, 31]. A recent in vitro study on colon cancer cells has proposed Ca^2+^-release-mediated p53-dependent and –independent pathways of KP46-mediated programmed cell death induction. While in p53^+/+^ cells p53-induced upregulation of reactive oxygen species (ROS) was proposed as central mode-of action downstream of intracellular Ca^2+^-release, in p53-mutated or -deleted cells FAS-related extrinsic apoptosis was postulated [31]. Accordingly, a p53-dependent cell death mechanism via KP46 accumulation in mitochondria and deregulation of mitochondrial dynamics and bioenergetics was suggested recently for human colon cancer cells [13]. Depletion of the intracellular labile iron pool by KP46 initiated p53-dependent BNIP3L activation and in turn mitophagic cell death [13]. However, in a previous study we were unable to detect any evidence for an impact of the p53 status on the general sensitivity of an extended panel of lung and colon cancer cells against KP46 treatment [17]. Accordingly, of the here investigated OS cell lines only U-2 OS harbors an intact p53 response while the other three cell models are p53 mutated or null [32, 33]. However, U-2 OS was characterized by the lowest sensitivity against KP46 arguing against a major sensitizing role of wild-type p53 against this gallium-containing metal compound. Consequently, though p53-mediated stress or damage recognition might impact on the mode of KP46-induced cell death, it is obviously not a general determinant of cancer cell sensitivity to KP46.

OS cells responded to KP46 by a rapid loss of cell adhesion and rounding up with a subpopulation of dead cells lacking classical features of apoptosis like chromatin condensation and formation of apoptotic bodies. Recently, our group has described a comparable anoikis-like cell death in KP46-hypersensitive colon and lung cancer cell models [17]. This additional form of KP46-induced cell death was insensitive to caspase-inhibitors but characterized by distinct downregulation of integrin β1 accompanied by cleavage of talin in a calpain inhibitor-sensitive fashion. Calpain regulates integrin turnover through degradation and cleavage of talin, the binding partner of integrin [34, 35]. Whether loss of integrin β1 expression, also detected in KP46-treated OS cells (data not shown), is the underlying mechanism is matter of ongoing investigations. Additionally to the rapid loss of cell adhesion, already low, subtoxic KP46 concentrations significantly blocked OS cell migration. As metastatic spread is still the leading cause of death for OS patients [2], these anti-adhesive and anti-migratory effects of KP46 might be especially interesting to develop this bone-targeting gallium compounds for systemic treatment of OS.

Combined chemotherapy has dramatically improved outcome of OS patients, however, relapse occurs is a certain percentage of cases based on resistance development. Hence, therapy response is a major prognostic factor for OS patient survival [27]. Consequently, we tested whether KP46 might represent a feasible candidate for anti-OS combination strategies. In our setting, synergistic effects were most pronounced when KP46 was used together with the standard OS agent cisplatin. Comparable observations have been reported in early studies for the platinum drugs cisplatin, carboplatin and oxaliplatin in ovarian and colon cancer cells [9]. The underlying molecular mechanisms of this synergism are currently enigmatic and might include interactions with repair mechanism or programmed cell death regulatory signals. Accordingly, we found upregulation of pro-apoptotic Bcl-2 family members Bax and Bim as well as enhanced Bax mitochondrial translocation in KP46-treated colon and lung cancer cells [17]. Pro- and anti-apoptotic Bcl-2 family members and especially expression of Bcl-2 and Mcl-1 are also decisive in the regulation of cisplatin sensitivity of OS cells [36, 37]. Consequently, we also combined two inhibitors of anti-apoptotic Bcl-2 family members, namely the BH3 mimetics obatoclax and venetoclax (ABT-199), with KP46. Unexpectedly, only the former drug, being a relatively broad Bcl-2 inhibitor, strongly synergized in the majority of OS cells with KP46, while the relatively Bcl-2-specific venetoclax failed to enhance the anti-OS activity. This argues against a major inhibitory function of anti-apoptotic Bcl-2 family members on KP46-induced OS cell death.

Additionally to Bcl-2 inhibition, obatoclax but not venetoclax is known to effectively block autophagy by interfering with autophagosomal acidification [28]. Especially in the less KP46-sensitive OS cell model U-2OS, KP46 clearly induced autophagy by appearance of larger acidic vesicles and accumulation of the processed form of LC3B, the phosphatidylethanolamine-bound LC3-II, a widely used autophagy marker. Combination experiments suggested an enhanced autophagic flux by KP46 which could be blocked by combination with downstream autophagy inhibitors obatoclax and chloroquine. Indeed, KP46-induced macroautophagy and mitophagy were also recently described in HCT116 cells, the latter representing a mode-of-action contributing to the cytotoxic activity [13]. Accordingly, mitophagy inhibition by downregulation of BNIP3L rescued cells from rapid KP46-mediated cell death. However, on the contrary, macroautophagy is a well-described resistance mechanism also of OS cells against a variety of synthetic and natural anticancer drugs [38, 39] suggesting that autophagy inhibition might synergize with KP46. Furthermore, there is evidence that the inhibition of integrin function and thus loss of cell adhesion, leads to protective autophagy induction and enables survival if cell re-adhesion is possible. Additionally, autophagy seems to be an important component in the life cycle of integrins and has a direct impact on cell migration [38, 40–44]. Therefore we hypothesized that autophagy inhibitors should sensitize OS cells to the anti-tumor activity of KP46. Indeed, not only obatoclax but also the classical late stage autophagy inhibitor chloroquine demonstrated comparable synergistic effects in the investigated OS cell lines with the exception of SAOS-2 cell, where the effects were additive. Together this strongly suggests that the inhibition of autophagic flux by obatoclax and not its interaction with bcl-2 family members is underlying the synergism with KP46. Consequently, at least in OS cells macro-autophagy is obviously a way to remove damages induced by KP46 to allow cancer cell survival.

## Conclusion

In this study, we demonstrate that the currently clinically tested and bone-targeting gallium compound KP46 is highly active against OS cell lines by inducing cancer cell death but also inhibiting their migratory potential. Autophagy induction was identified to protect OS cells against KP46-mediated damage and hence combination with autophagy inhibitors exerts markedly synergistic effects. Additionally, also cisplatin, another autophagy inducer in OS cells [45], but - in contrast to KP46 - mainly targeting DNA - clearly synergizes with the gallium compound. Whether this combination might synergistically enhance autophagy induction turning this protective mechanism into autophagic cell death is matter of ongoing investigations. Together this implies that the bone-targeting gallium compound KP46 might bear promising potential for treatment of OS and may be effective as single agent or in combination with standard chemotherapy or compounds inhibiting autophagy. The respective in vivo analyses have already been initiated.

## Additional files

Additional file 1: Figure S1. Rapid morphological changes induced by short-term treatment of OS cells with KP46. HOS and SAOS-2 cells were treated with the indicated concentrations of KP46 for 24 h and microphotographs taken at phase contrast settings. Size bar, 50 μm. (PDF 295 kb)

Additional file 2: Figure S2. Induction of large acidic vesicles by KP46 in OS cells and impact of obatoclax. (A) HOS cells were treated with KP46 alone or in combination with obatoclax as indicated. Photomicrographs were taken after 48 h drug exposure in phase contrast setting. Size bar, 10 μm. (B) HOS cells treated with KP46 for 24 h at the indicated concentrations in combination with obatoclax and stained with AO. Fluorescence images were taken with FITC (green) and TRITC (red) filter sets. Size bar, 10 μm. For the respective KP46 single agent photomicrographs compare Fig. 5a. (PDF 353 kb)

Additional file 3: Figure S3. KP46 treatment synergizes with the autophagy inhibitor chloroquine. Cell viability after combined treatment of OS cells with KP46 and chloroquine for 72 h at the indicated concentrations was determined by MTT survival assay. CI values for HOS and SAOS-2 cells derived from two independent experiments in triplicate are shown representatively. The respective growth curves are shown in Fig. 5d. (PDF 160 kb)

## Abbreviations

AO: Acridine orange
ATTC: American type culture collection
CI: Combination index
FCS: Fetal calf serum
HLF: Human lung fibroblast
HOE/PI: Hoechst/propidium iodide
LC3II: Microtubule-associated protein light chain 3II
MTX: Methotrexate
OS: Osteosarcoma
ROS: Reactive oxygen species
