# Supplementary figures and images for: Distinct activity of the bone-targeted gallium compound KP46 against osteosarcoma cells - synergism with autophagy inhibition
Source: J Exp Clin Cancer Res. 2017 Apr 12;36:52. doi: 10.1186/s13046-017-0527-z (PMC5389188; doi:10.1186/s13046-017-0527-z)

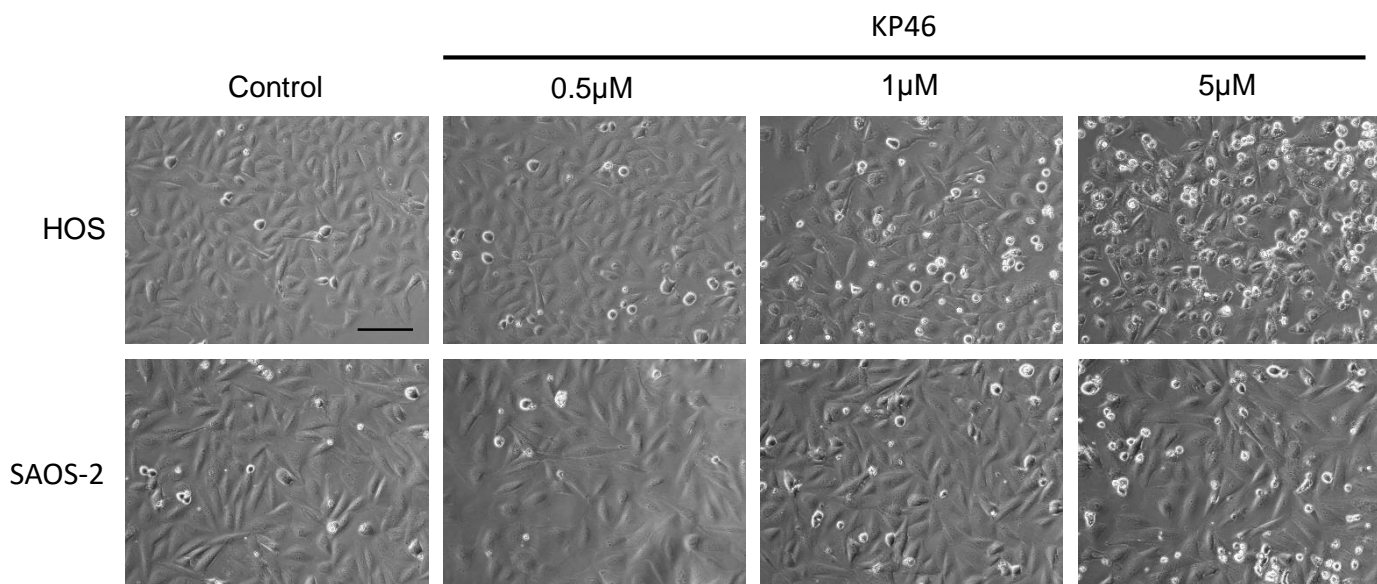

Additional file 1 Figure S-1

Supplement: Supplementary file 1 — Rapid morphological changes induced by short-term treatment of OS cells with KP46. HOS and SAOS-2 cells were treated with the indicated concentrations of KP46 for 24 h and microphotographs taken at phase contrast settings. Size bar, 50 μm. (PDF 295 kb) [file 13046_2017_527_MOESM1_ESM.pdf]

A

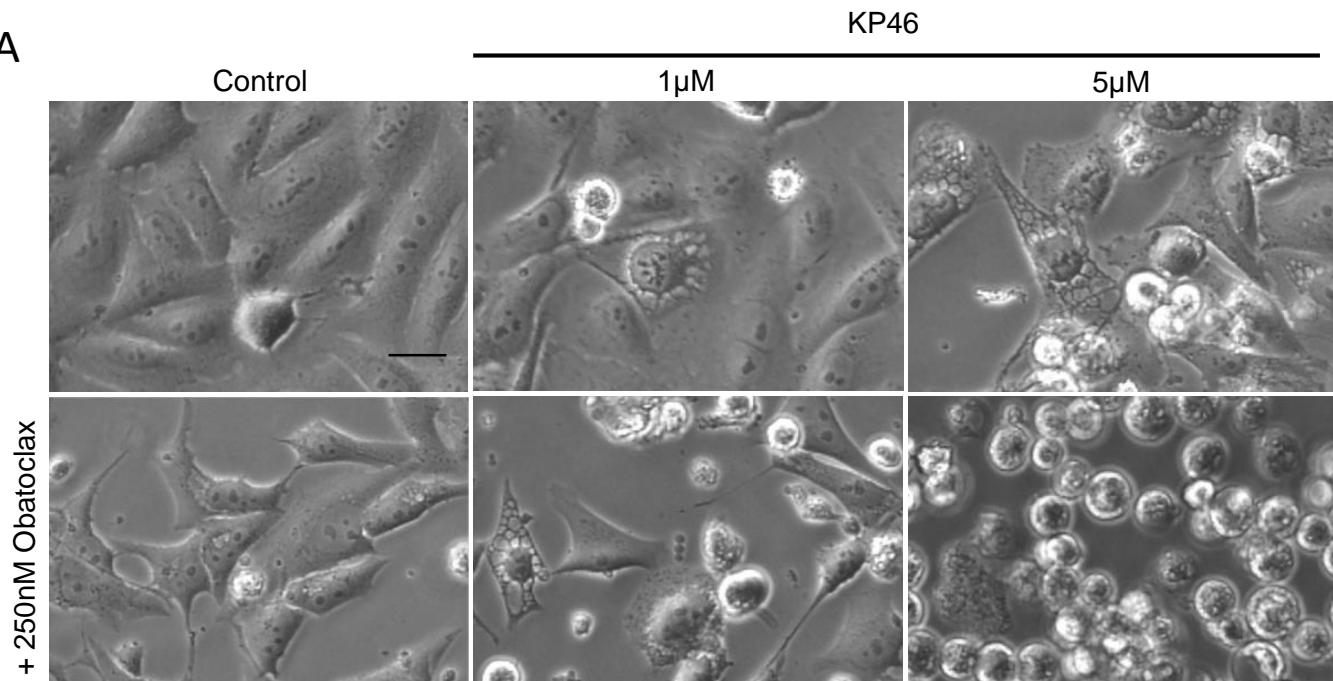

B

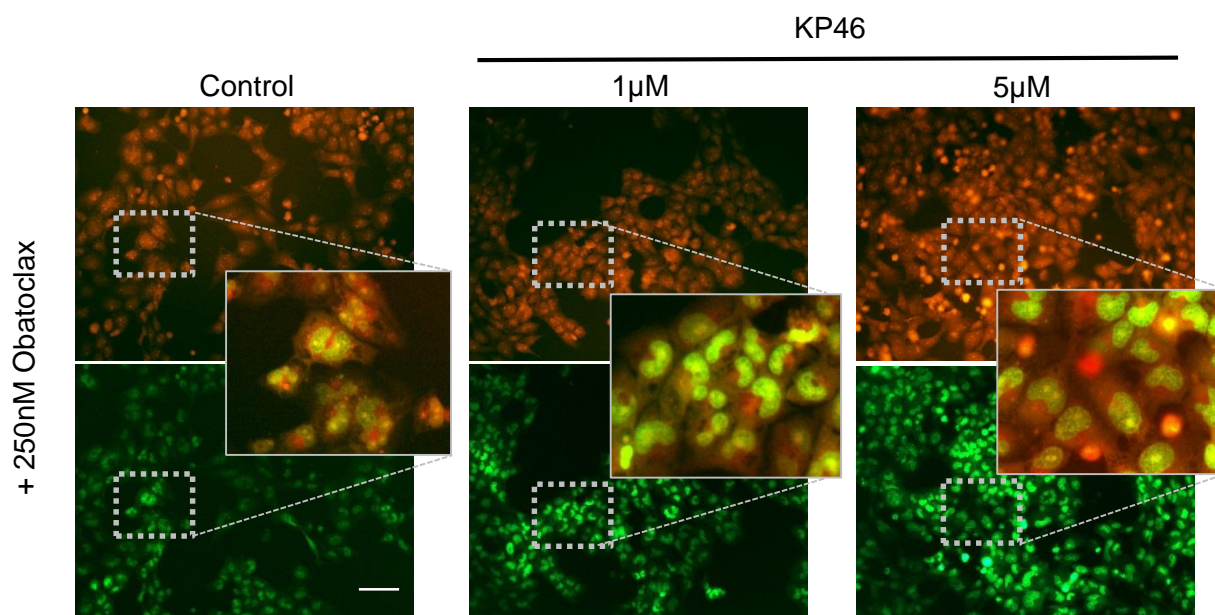

Supplement: Supplementary file 2 — Induction of large acidic vesicles by KP46 in OS cells and impact of obatoclax. (A) HOS cells were treated with KP46 alone or in combination with obatoclax as indicated. Photomicrographs were taken after 48 h drug exposure in phase contrast setting. Size bar, 10 μm. (B) HOS cells treated with KP46 for 24 h at the indicated concentrations in combination with obatoclax and stained with AO. Fluorescence images were taken with FITC (green) and TRITC (red) filter sets. Size bar, 10 μm. For the respective KP46 single agent photomicrographs compare Fig. 5a. (PDF 353 kb) [file 13046_2017_527_MOESM2_ESM.pdf]

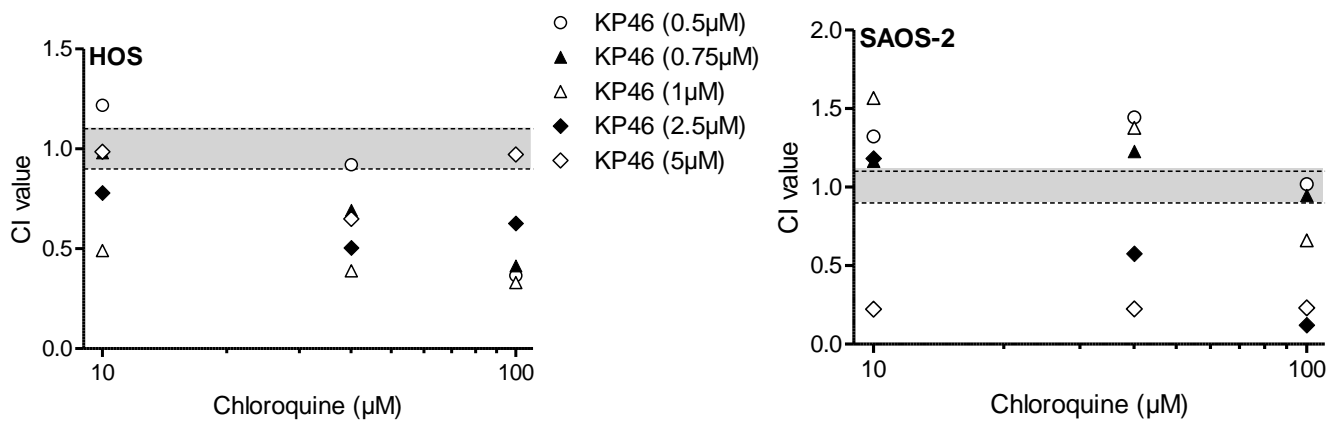

Supplement: Supplementary file 3 — KP46 treatment synergizes with the autophagy inhibitor chloroquine. Cell viability after combined treatment of OS cells with KP46 and chloroquine for 72 h at the indicated concentrations was determined by MTT survival assay. CI values for HOS and SAOS-2 cells derived from two independent experiments in triplicate are shown representatively. The respective growth curves are shown in Fig. 5d. (PDF 160 kb) [file 13046_2017_527_MOESM3_ESM.pdf]
